# Supplementary material for: FBXW2 suppresses migration and invasion of lung cancer cells via promoting β-catenin ubiquitylation and degradation
Source: Nat Commun. 2019 Mar 27;10:1382. doi: 10.1038/s41467-019-09289-5 (PMC6437151; doi:10.1038/s41467-019-09289-5)
Supplement: Supplementary file 1 — Supplementary Information [file 41467_2019_9289_MOESM1_ESM.pdf]

Supplementary Information

FBXW2 suppresses migration and invasion of lung cancer cells via promoting β-catenin ubiquitylation and degradation

Yang et al.

Table of Contents

**Supplementary Figure 1.**	**FBXW2 binds to β-catenin.**
**Supplementary Figure 2.**	**FBXW2 reduces β-catenin levels and transcriptional activity.**
**Supplementary Figure 3.**	**FBXW2 ubiquitylates β-catenin and shortens its half-life.**
**Supplementary Figure 4:**	**AKT1 is required for FBXW2-mediated β-catenin degradation.**
**Supplementary Figure 5.**	**FBXW2 suppresses the migration and invasion in vitro and in vivo.**
**Supplementary Figure 6.**	**AKT1-FBXW2-β-catenin axis is specific for migration and invasion.**
**Supplementary Figure 7.**	**β-catenin^{S552} induces MMPs to mediate migration and invasion.**
**Supplementary Figure 8.**	**β-catenin expression in xenograft tumors.**
**Supplementary Figure 9.**	**Gel source images.**
**Supplementary Table 1.**	**List of primers used for mutagenesis.**
**Supplementary Table 2.**	**List of siRNAs and shRNAs.**
**Supplementary Table 3.**	**List of primers used for ChIP qPCR.**
**Supplementary Table 4.**	**List of primers used for real-time qPCR.**

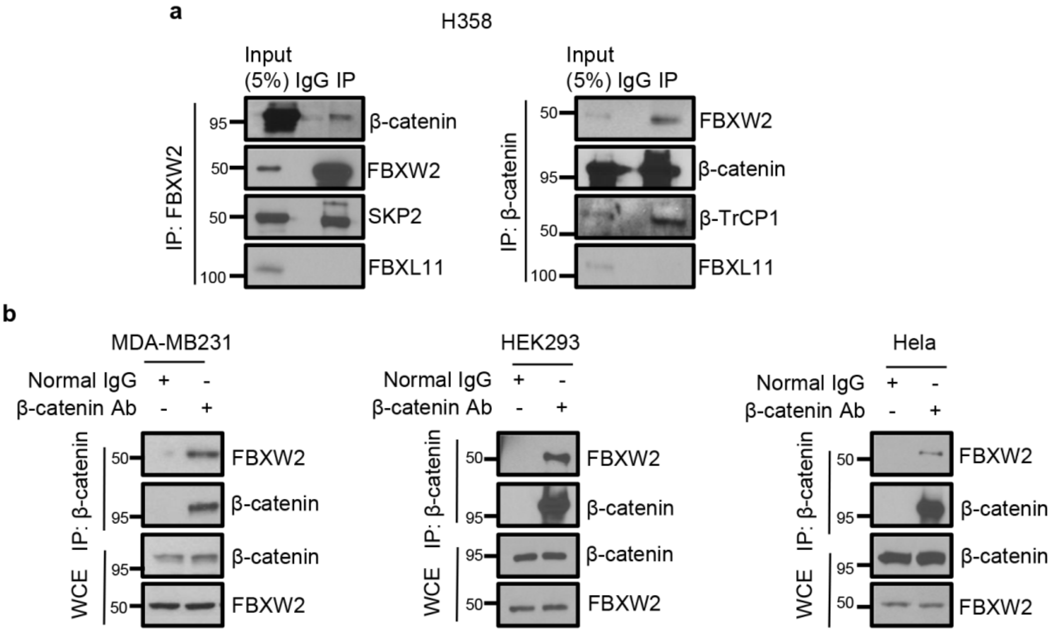

Supplementary Figure 1. FBXW2 binds to β-catenin. (a and b) The interaction between endogenous FBXW2 and endogenous β-catenin in multiple cell lines: Cell lysates from H358 (a), MDA-MB231, HEK293, and Hela cells (b) were pulled down with anti-FBXW2 or anti-β-catenin Abs, followed by IB with indicated Abs. WCE: whole cell extract. Unprocessed original scans of blots are shown in Supplementary Figure 9.

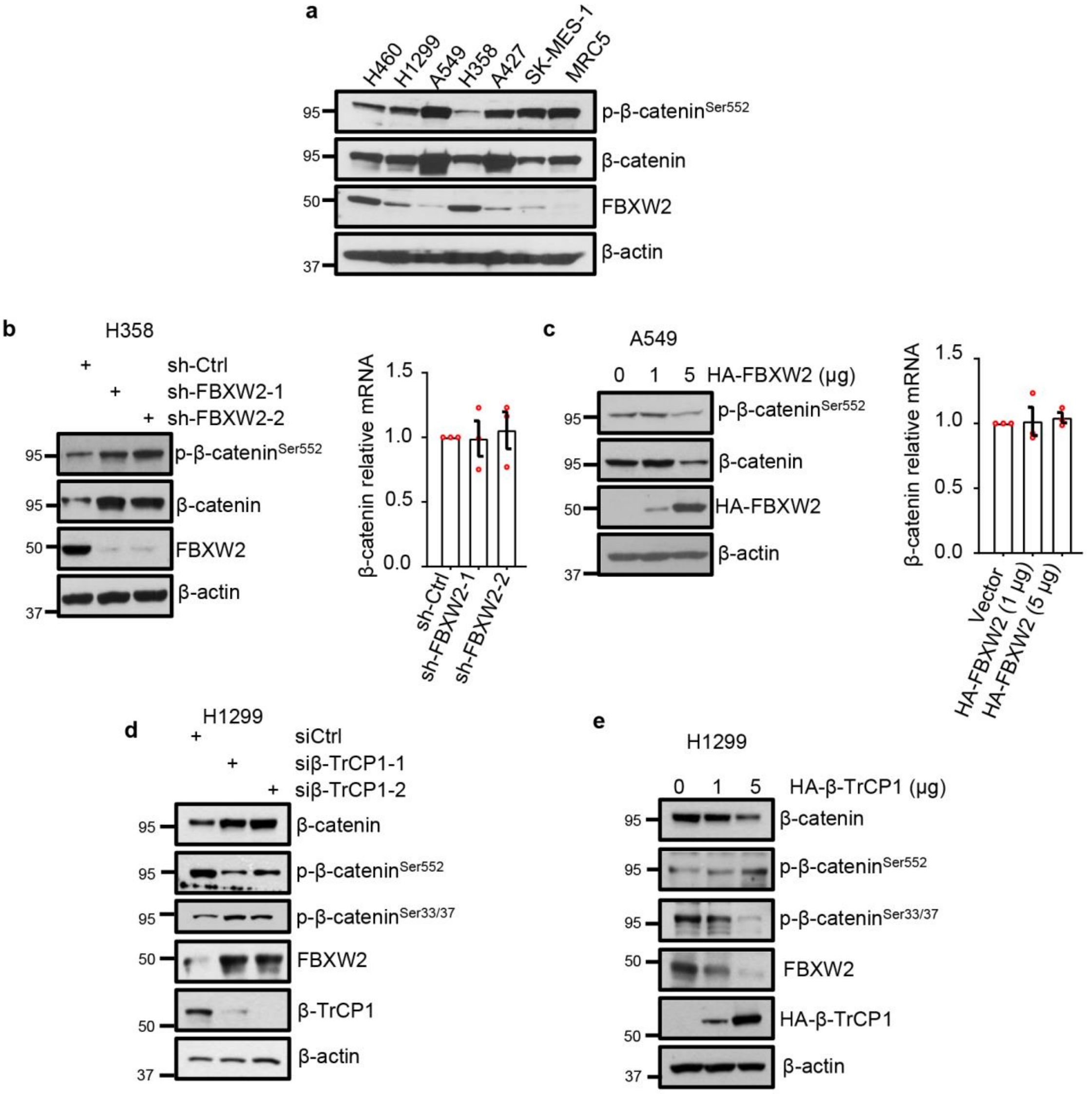

Supplementary Figure 2. FBXW2 reduces β-catenin levels and transcriptional activity. (a) Inverse correlation between the protein levels of FBXW2 and β-catenin in multiple lung cancer cell lines: Human lung cancer cells were harvested for IB with indicated Abs. **(b)** FBXW2 silencing increases the endogenous protein levels of β-catenin, but has no effect on its mRNA levels: H358 cells were infected with lentivirus expressing indicated shRNA, followed by IB (left) or qRT-PCR (right). Data are shown as mean ±s.e.m. of three independent experiments. **(c)** FBXW2 overexpression reduces β-catenin protein levels, but has no effect on its mRNA levels: A549 cells were transfected with increasing amounts of FBXW2, followed by IB with indicated Abs (left) or qRT-PCR (right). Data are shown as mean ±s.e.m. of three independent experiments. **(d)** β-TrCP knockdown increases the endogenous protein levels of total β-catenin as well as phosphorylated β-catenin-Ser$^{33/37}$, but decreases phosphorylated β-catenin-Ser^{552}: H1299 cells were transfected with siRNA oligonucleotide targeting β-TrCP, followed by IB. **(e)** β-TrCP overexpression reduces phosphorylated β-catenin-Ser$^{33/37}$ and total β-catenin protein levels, but increases β-catenin-Ser^{552} protein level: H1299 cells were transfected with increasing amounts of β-TrCP, followed by IB with indicated Abs. Unprocessed original scans of blots are shown in Supplementary Figure 9.

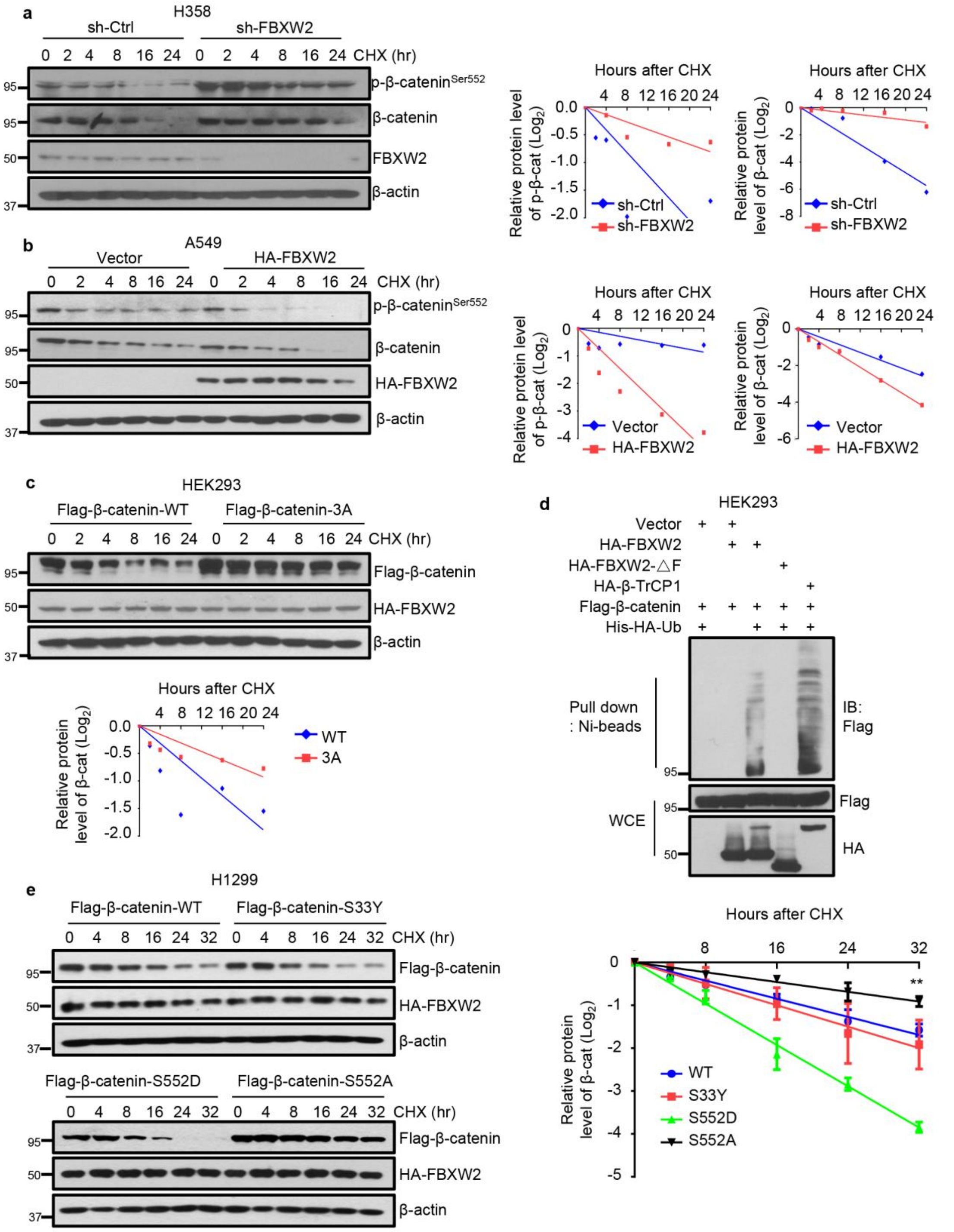

Supplementary Figure 3. FBXW2 ubiquitylates β-catenin and shortens its half-life. **(a)** FBXW2 silencing extends the protein half-life of both phospho-β-catenin and total β-catenin: H358 cells were infected with lentivirus expressing shRNA targeting FBXW2 or scrambled control shRNA. Cells were then treated with CHX for indicated time periods, followed by IB with indicated Abs (left). The band density was quantified using ImageJ software and plotted (right). **(b)** Overexpression of FBXW2 shortens the protein half-lives of both phospho-β-catenin and total β-catenin: A549 cells were transfected with HA-FBXW2 or mock vector for 48h, and then treated with CHX for indicated time periods, followed by IB with indicated Abs (left). The band density was quantified using

ImageJ software and plotted (right). **(c)** FBXW2 shortens the protein half-life of β-catenin-WT protein, but not β-catenin-3A: HEK293 cells were transfected with indicated plasmids for 48h, and then treated with CHX for indicated time periods, followed by IB with indicated Abs (top). The band density was quantified using ImageJ software and plotted (bottom). **(d)** FBXW2, but not FBXW2-ΔF mutant promotes β-catenin ubiquitylation *in vivo*: HEK293 cells were transfected with indicated plasmids, followed by pull-down using Ni-NTA beads (top) or direct IB with indicated Abs (bottom). **(e)** β-catenin-S552A is more stable in EGF stimulated cells than β-catenin-WT/S33Y/S552D: H1299 cells were transfected with indicated plasmids for 24h, then serum starved for 24 h, followed by the addition of serum and EGF (100 ng/ml) in the presence of CHX for indicated time periods. Cells were then harvested for IB with indicated Abs (left). The band density was quantified using ImageJ software and plotted (right), ** $p < 0.01$ (One-way ANOWA). Unprocessed original scans of blots are shown in Supplementary Figure 9.

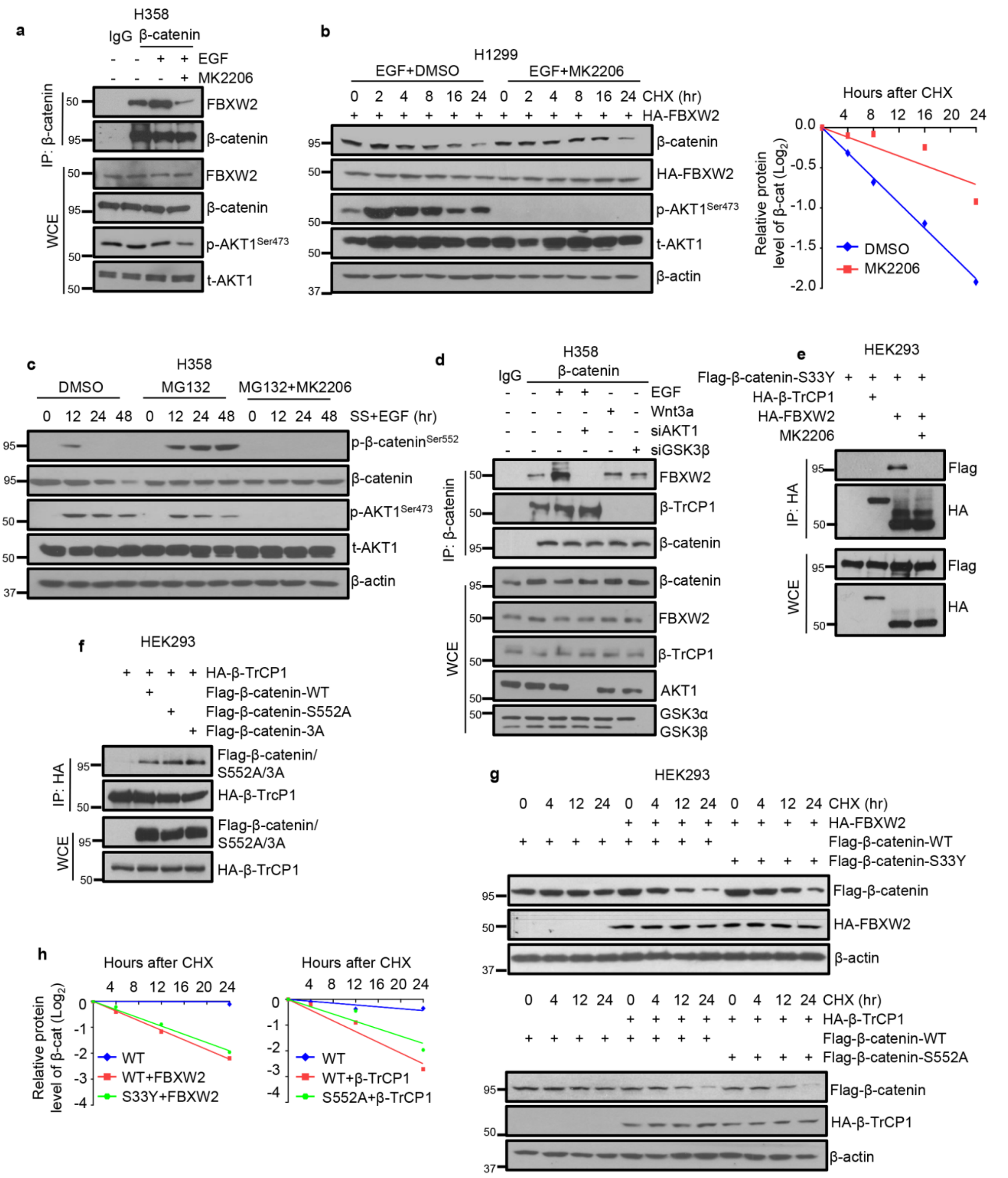

Supplementary Figure 4. AKT1 is required for FBXW2-mediated β-catenin degradation. (a) AKT inhibitor MK2206 inhibits FBXW2-β-catenin interaction: H358 cells were pretreated with AKT inhibitor MK2206 (3 μM or 10 μM) for 2h, followed by the treatment of EGF (100 ng/ml) and MG132 (10 μM) for additional 6 h. Cells were then harvested, followed by IP with β-catenin Ab and IB with indicated Abs. **(b)** AKT inhibitor MK2206 extends β-catenin protein half-life: H1299 cells were transfected with HA-FBXW2 or mock vector for 48h, followed by pretreatment with AKT inhibitor MK2206 (3 μM or 10 μM) and EGF (100 ng/ml) for 2h. Cells were then treated with CHX for indicated time periods before being harvested for IB (left). The band density was quantified using ImageJ software and plotted (right). **(c)** Proteasome inhibitor MG132 abrogates AKT1-dependent β-catenin degradation: H358 cells were serum starved for 24 h, followed by the addition serum and EGF (100 ng/ml) in the absence or

presence of MG132 (1 μM) and MK2206 (1 μM). Cells were then harvested at indicated time points for IB with indicated Abs. **(d)** The FBXW2-β-catenin binding is enhanced by EGF treatment, blocked by AKT1 silencing, but is not affected by Wnt3a treatment, nor by GSK3β silencing: H358 cells were transfected with siRNA targeting AKT1 or GSK3β, followed by the treatment with EGF (100 ng/ml) or Wnt3a (100 ng/ml) for 6 h. Cells were then treated with MG132 for the last 2 h before being harvested for IP with β-catenin Ab and IB with indicated Abs. **(e)** S33Y mutant binds to FBXW2, which can be blocked by MK2206: HEK293 cells were transfected with indicated plasmids, and then treated with MK2206 (3 μM) and MG132 (10 μM) for 8 h, followed by IP with anti-HA Ab and IB with indicated Abs. **(f)** β-TrCP1 binds to β-catenin-S552A and β-catenin-3A as to β-catenin-WT: HEK293 cells were transfected with indicated plasmids, followed by IP with anti-HA Ab and IB with indicated Abs. **(g and h)** FBXW2 shortens the half-lives of β-catenin-WT and β-catenin-S33Y, while β-TrCP1 shortens the half-lives of β-catenin-WT and β-catenin- S552A: HEK293 cells were transfected with indicated plasmids for 48 h, and then treated with CHX for indicated time periods, followed by IB with indicated Abs (g). The band density was quantified using ImageJ software and plotted (h). Unprocessed original scans of blots are shown in Supplementary Figure 9.

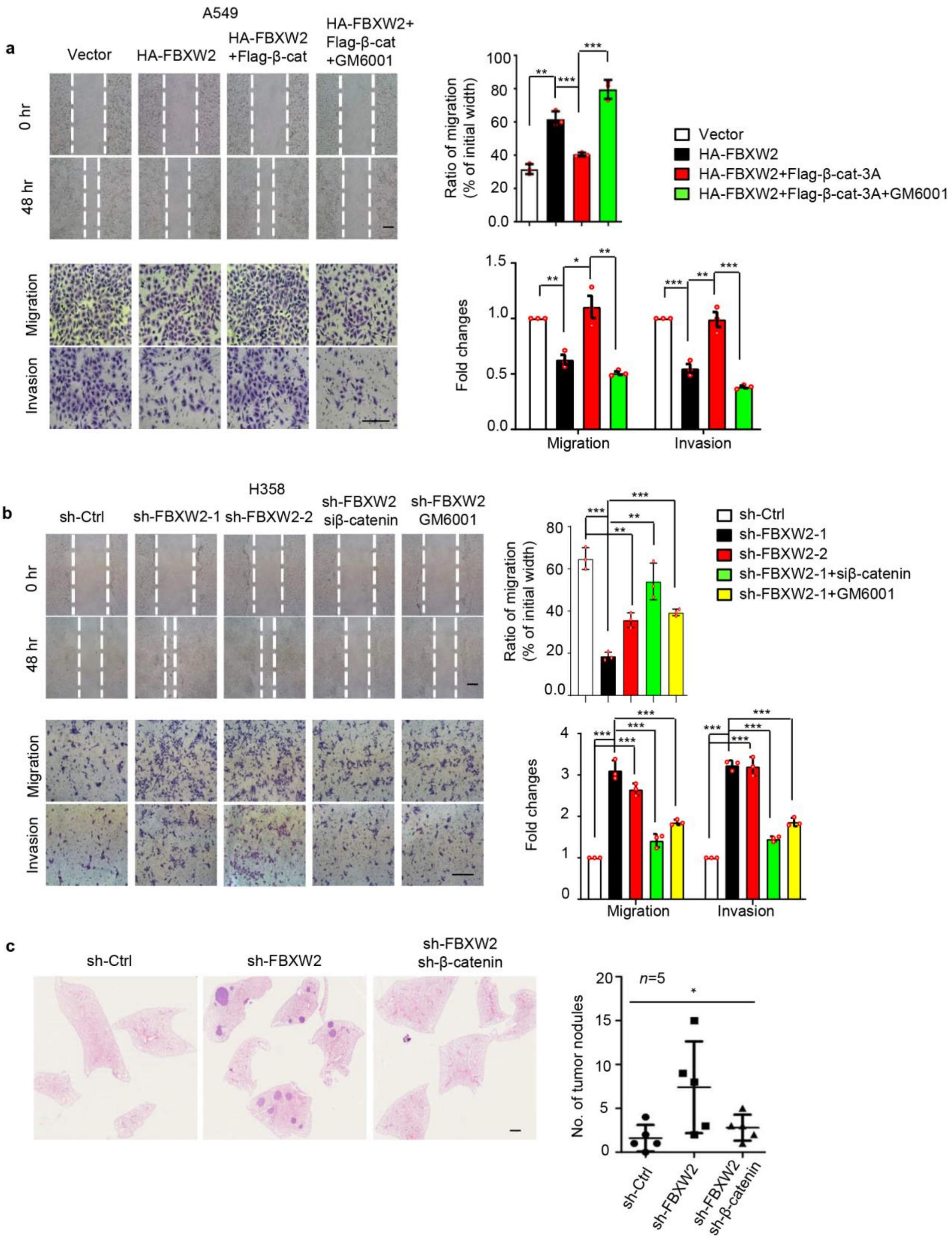

Supplementary Figure 5. FBXW2 suppresses the migration and invasion *in vitro* and *in vivo*. (a) Ectopic expression of FBXW2 suppresses cell migration and invasion, which can be blocked by simultaneous transfection of β-catenin in a MMPs-dependent manner:**(a)** A549 cells stably expressing indicated constructs selected by G418were treated with DMSO or GM6001 (5 μM), followed by wound healing assay (top) and transwell chamber migration and invasion assays (bottom). Shown are representative images of migrated cells in the wound healing assay (top, left) and in the transwell chamber migration and invasion assays (bottom, left). The ratios of migration were

calculated by dividing the width of the wound after 24 h and the width of initial wound (top, right). The number of migrated cells was counted in five random fields per chamber and statistically analyzed (bottom, right). Data are shown as mean±s.e.m. of three independent experiments. * $p < 0.05$, ** $p < 0.01$, *** $p < 0.001$, (Student's *t* test). Scale bars, 100 μm. **(b)** FBXW2 knockdown stimulates cell migration and invasion, which is abrogated by simultaneous silencing of β-catenin or GM6001 treatment: H358 cells stably expressing shRNA targeting FBXW2 or scrambled control shRNA selected by puromycin were transfected with siRNA targeting β-catenin or scrambled control siRNA, or treated by DMSO or GM6001 (5 μM) followed by wound healing assay (top) and transwell chamber migration and invasion assays (bottom). Shown are representative images of migrated cells (left panels). The ratios of migration were calculated by deviding the width of the wound after 24 h and the width of initial wound (top, right). The number of migrated cells was counted in five random fields per chamber and statistically analyzed (bottom, right). Data are shown as mean±s.e.m. of three independent experiments. ** $p < 0.01$, *** $p < 0.001$, (Student's *t* test). Scale bars, 100 μm. **(c)** FBXW2 knockdown stimulates metastasis of lung cancer cells *in vivo*: H358 cells with stable FBXW2 knockdown alone or in combination with stable β-catenin knockdown were injected into nude mice via tail vein. After 8 weeks, mice were sacrificed and lung tissues were stained with hematoxylin and eosin and photographed (left). The number of lung metastasis nodules in all five lobes of the lung from each mouse was counted and statistically analyzed (right). $n = 5$ for each group, * $p < 0.05$ (One-way ANOWA). Scale bars, 1 mm.

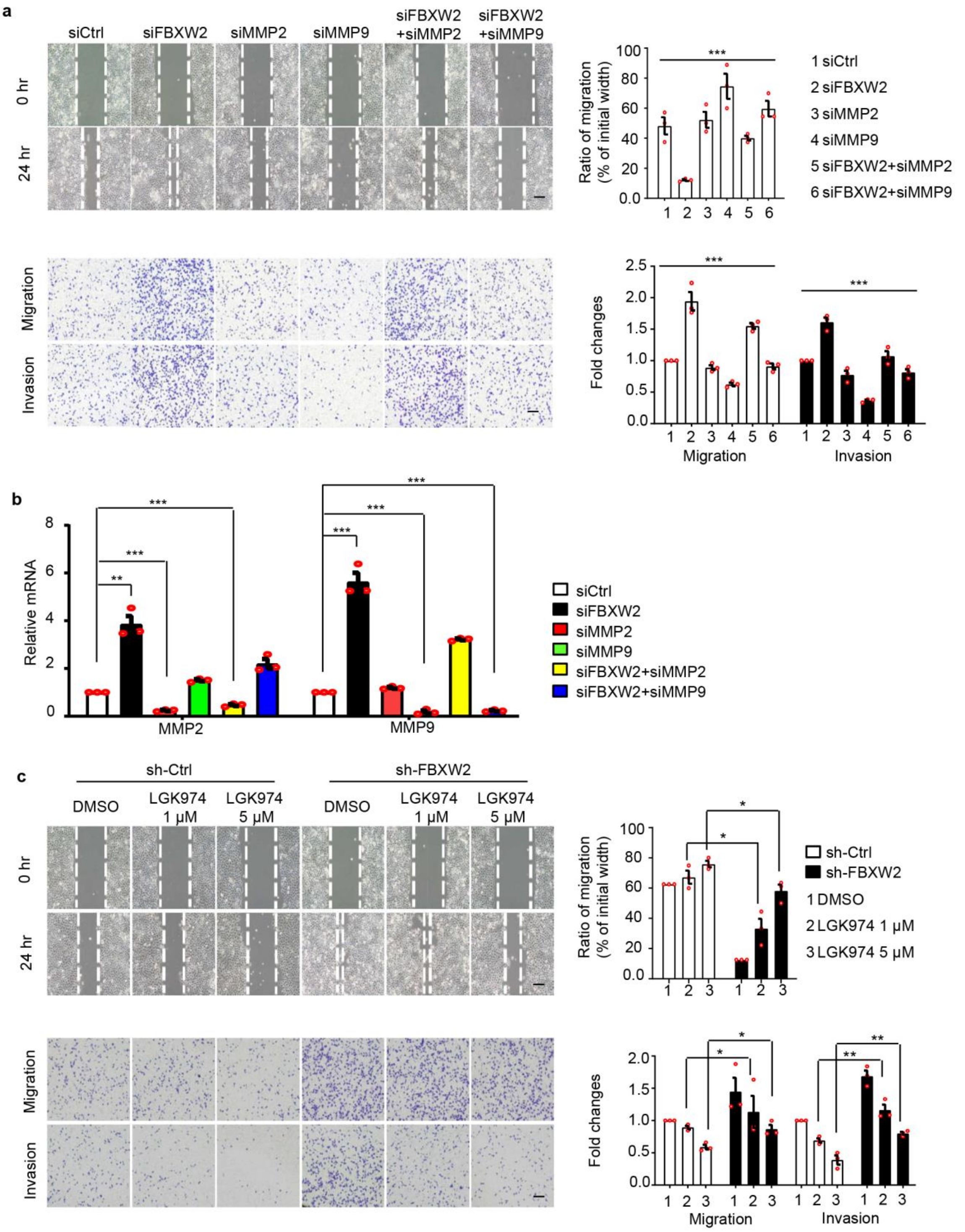

Supplementary Figure 6. AKT1-FBXW2-β-catenin axis is specific for migration and invasion. (a) FBXW2 knockdown stimulates cell migration and invasion, which is abrogated by simultaneous knockdown of MMP2 and MMP9: H1299 cells were transfected with the indicated siRNA, followed by wound healing assay (top) and transwell chamber migration and invasion assays (bottom). Shown are representative images of migrated cells (left panels). The ratios of migration were calculated by dividing the width of the wound after 24 h by the width of initial wound (top, right). The number

of migrated cells was counted in three random fields per chamber and statistically analyzed (bottom, right). Data are shown as mean±s.e.m. of three independent experiments, *** $p < 0.001$ (One-way ANOWA). Scale bars, 100 μm. **(b)** Real-Time PCR assays were performed in H1299 cells transfected with indicated siRNA. Data are shown as mean±s.e.m. of three independent experiments, ** $p < 0.01$, *** $p < 0.001$ (Student's *t* test). **(c)** Wnt signal inhibitor LGK974 partially rescued FBXW2 effect: H1299 cells were infected with the indicated lentiviral shRNA constructs, then treated with LGK974 1 μM or 5 μM for 24 h. The ratios of migration were calculated by dividing the width of the wound after 24 h and the width of initial wound (top, right). The number of migrated cells was counted in three random fields per chamber and statistically analyzed (bottom, right). Data are shown as mean±s.e.m. of three independent experiments, * $p < 0.05$, ** $p < 0.01$, (Student's *t* test). Scale bars, 100 μm.

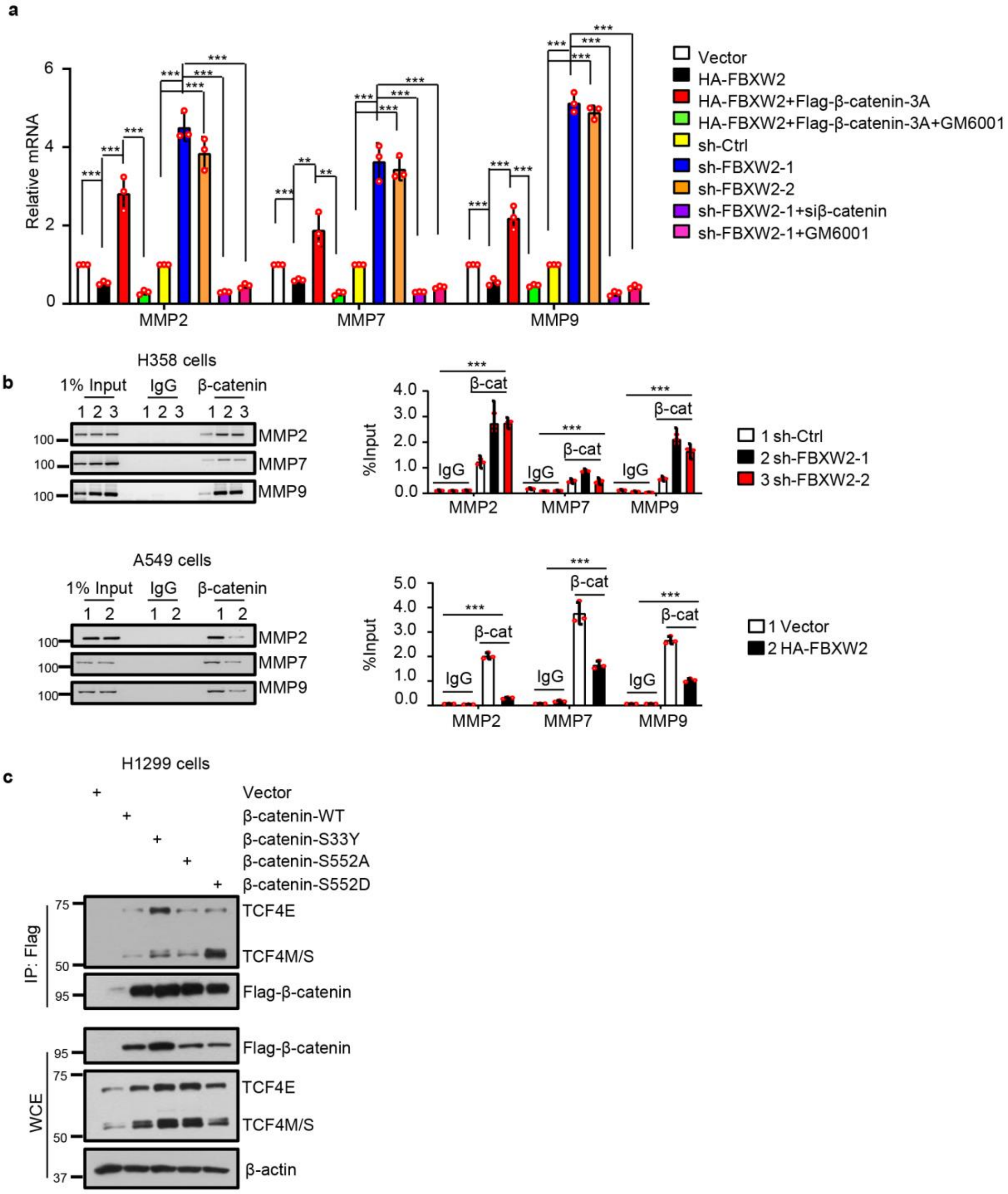

Supplementary Figure 7. β-catenin^{S552} induces MMPs to mediate migration and invasion. (a) FBXW2 negatively regulates the expression of MMPs, which is rescued by manipulation of β-catenin expression or using GM6001: H358 or A549 cells were transfected with indicated plasmids or infected with indicated lentivirus, and then left untreated or treated with GM6001, followed by qRT-PCR analysis. Data are shown as mean ± s.e.m. of three independent experiments. ** $p < 0.01$, *** $p < 0.001$(Student's *t* test). **(b)** FBXW2 negatively regulates β-catenin binding to the promoters of MMP genes: H358 cells were infected with lentivirus expressing indicated shRNA or scrambled control shRNA (top panels). A549 cells were transfected with mock vector or HA-FBXW2 (bottom panels). Cells were then harvested for ChIP assays with anti-β-catenin antibody. Mouse IgG was used as a negative control. Data are shown as mean ± s.e.m. of three independent experiments. *** $p < 0.001$(One-way ANOWA). **(c)** Differential binding of β-catenin mutants to TCF4 isoforms: H1299 cells were transfected

with indicated plasmids, followed by IP with anti-Flag beads and IB with indicated Abs. WCE: whole cell extract. Unprocessed original scans of blots are shown in Supplementary Figure 9.

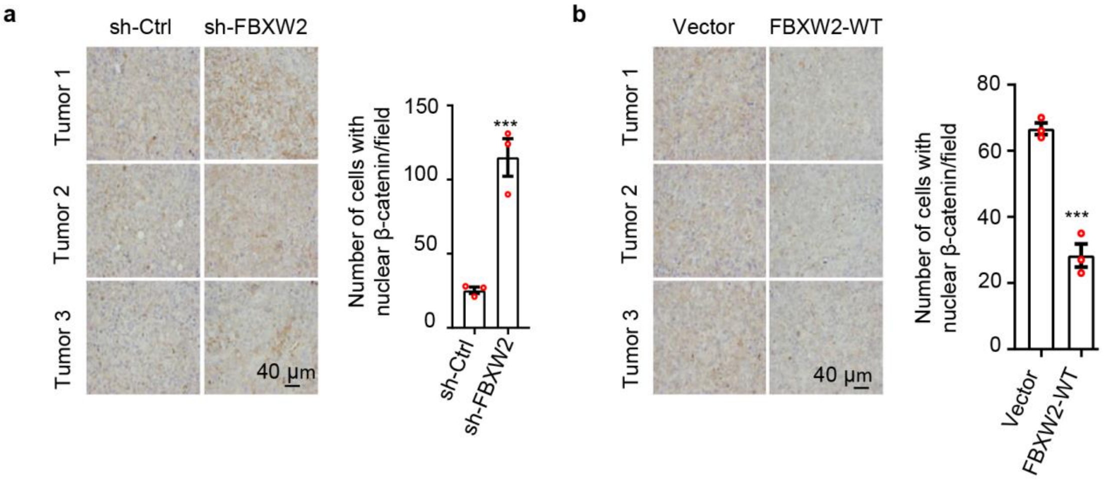

Supplementary Figure 8. β-catenin expression in xenograft tumors.
(**a and b**) β-catenin levels are increased in FBXW2 knockdown, but decreased in wild type FBXW2 overexpressed tumors: 1×10^6 cells H1299 cells stably expressing shRNA targeting FBXW2 or scramble control shRNA (a), or stably expressing wild-type FBXW2 (WT-FBXW2) were inoculated s.c. in both flanks of nude mice (b). After 8 weeks, xenograft tumors were harvested, followed by IHC staining with anti-β-catenin antibodies. Representative staining pictures of three tumors are shown (left panels). The number of cells with positive staining of β-catenin in nucleus was counted in three random fields per tumor and statistically analyzed (right panels). Data are shown as mean ± s.d. *** $p < 0.001$ (Student's *t* test).

Supplementary Figure 9. Gel source images

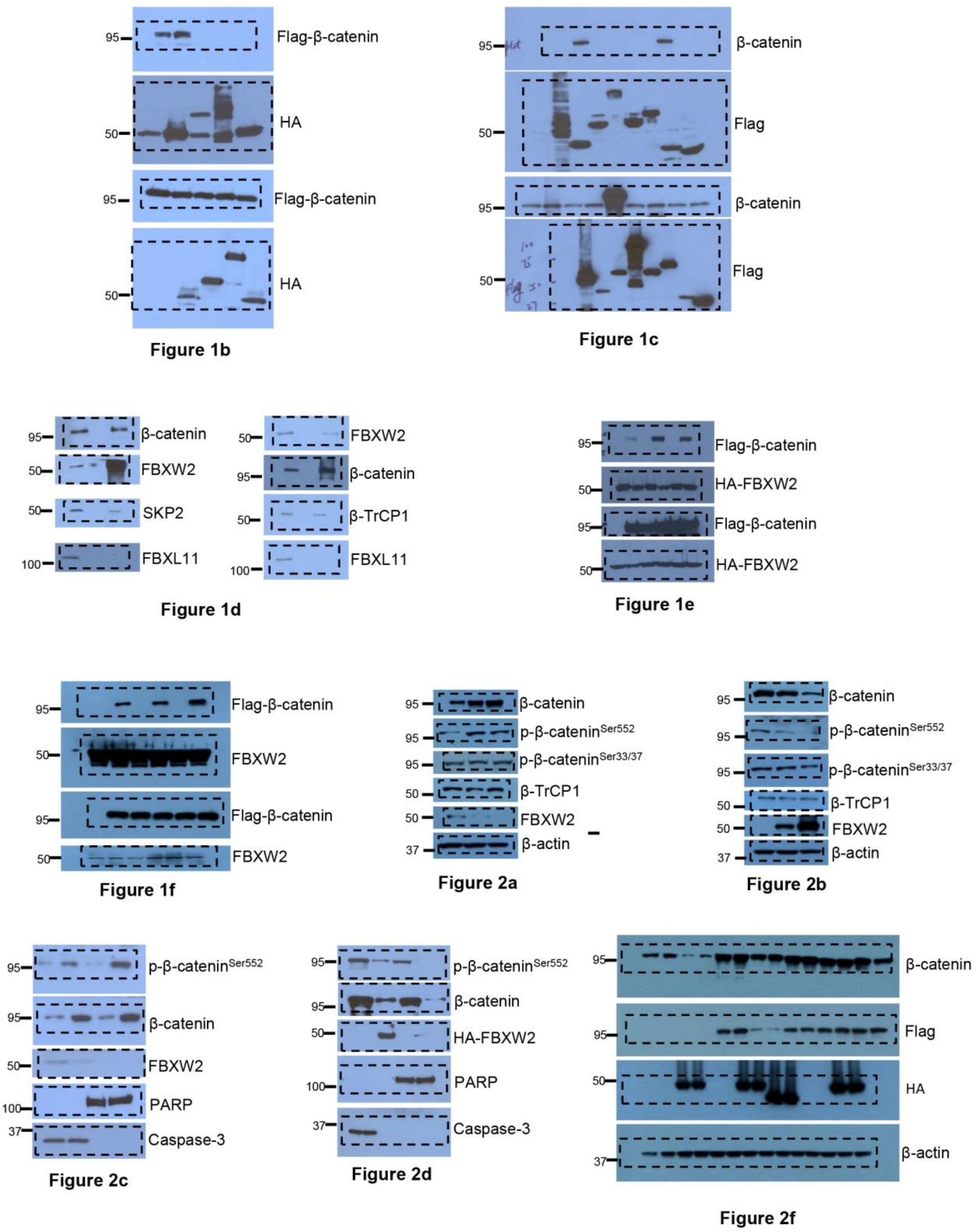

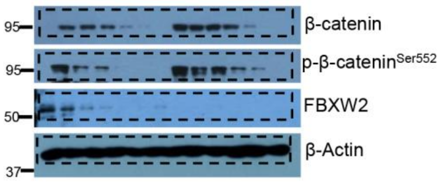

Figure 3a

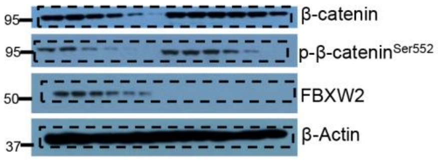

Figure 3b

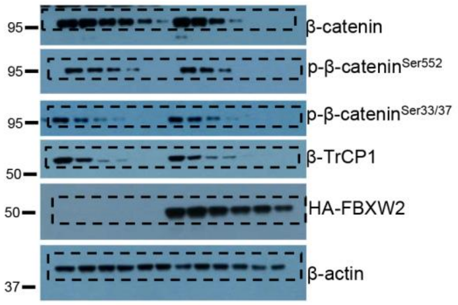

Figure 3c

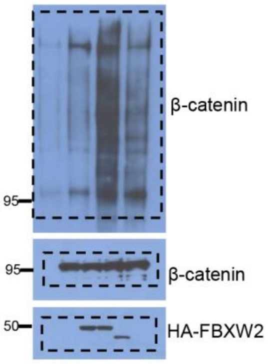

Figure 3d

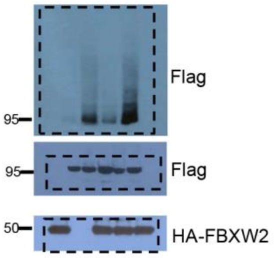

Figure 3e

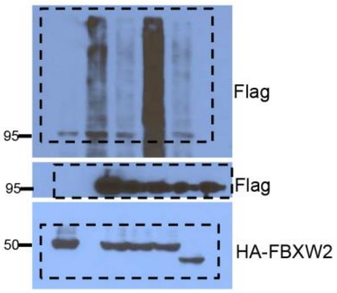

Figure 3f

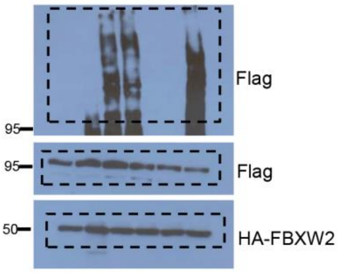

Figure 3g

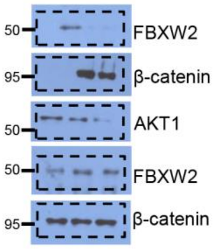

Figure 4a

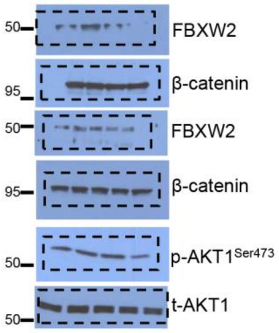

Figure 4b

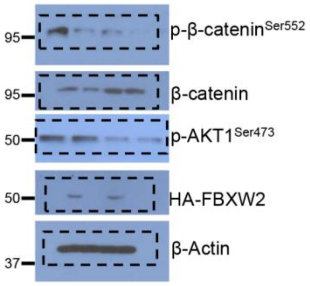

Figure 4c

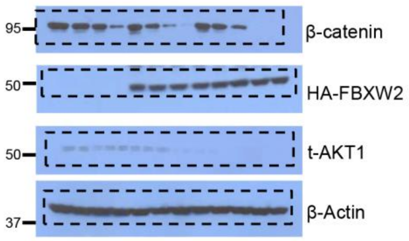

Figure 4d

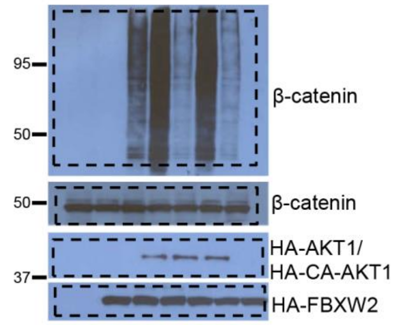

Figure 4f

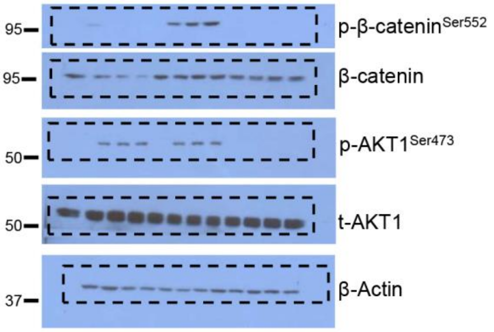

Figure 4g

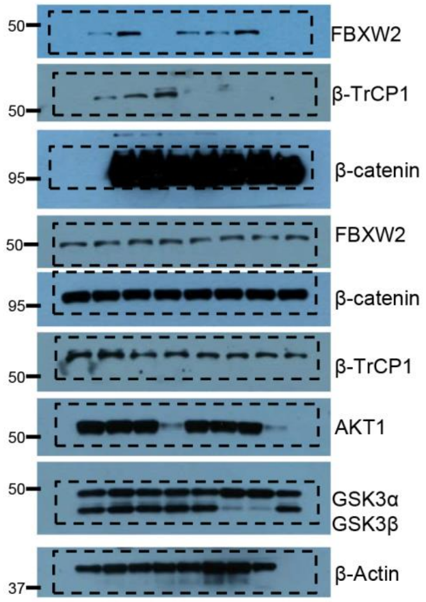

Figure 4h

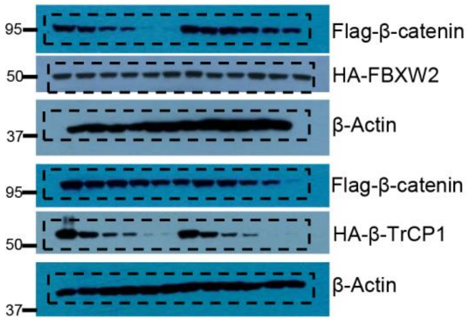

Figure 4i

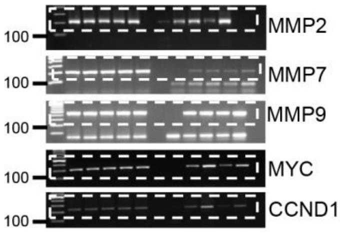

Figure 6d

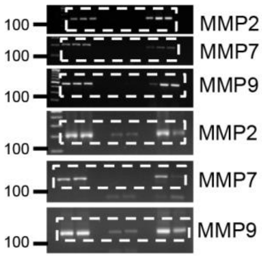

Figure 6e

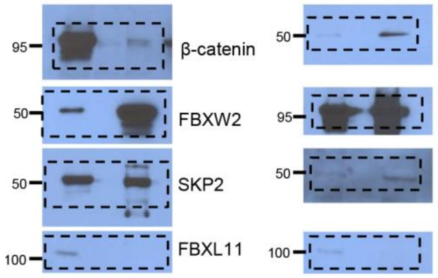

Supplementary Figure 1a

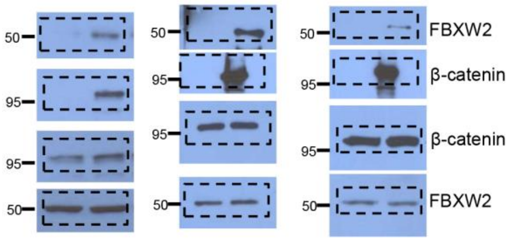

Supplementary Figure 1b

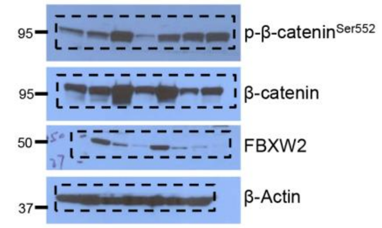

Supplementary Figure 2a

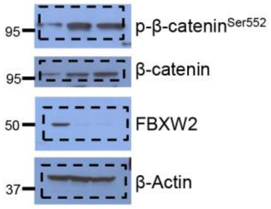

Supplementary Figure 2b

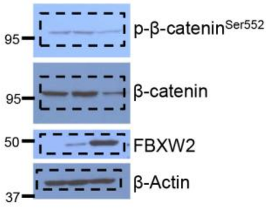

Supplementary Figure 2c

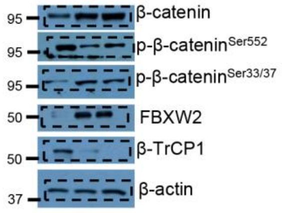

Supplementary Figure 2d

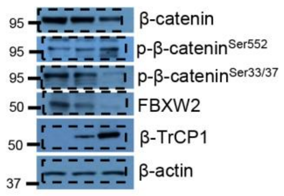

Supplementary Figure 2e

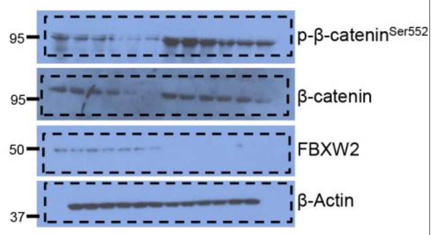

Supplementary Figure 3a

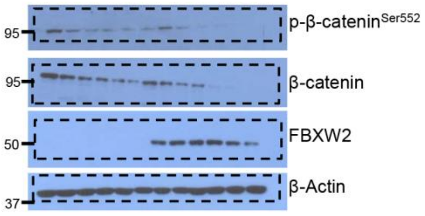

Supplementary Figure 3b

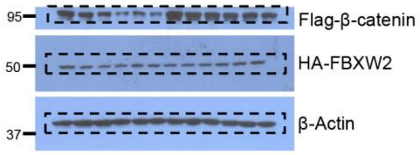

Supplementary Figure 3c

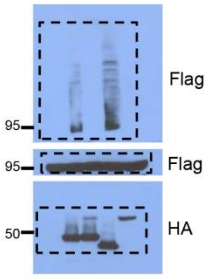

Supplementary Figure 3d

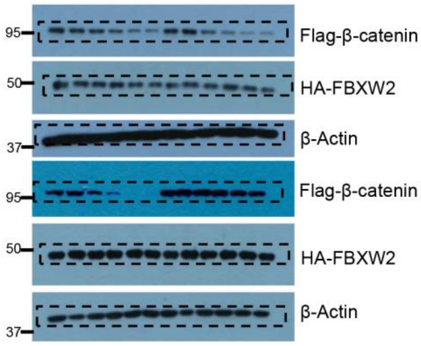

Supplementary Figure 3e

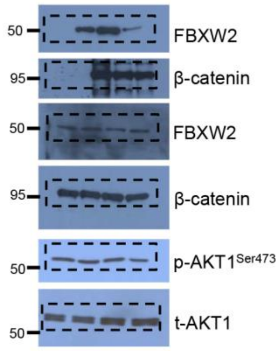

Supplementary Figure 4a

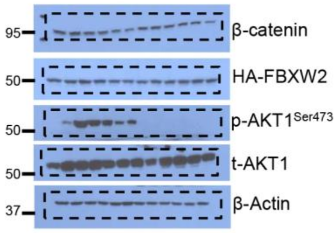

Supplementary Figure 4b

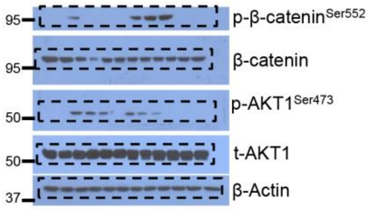

Supplementary Figure 4c

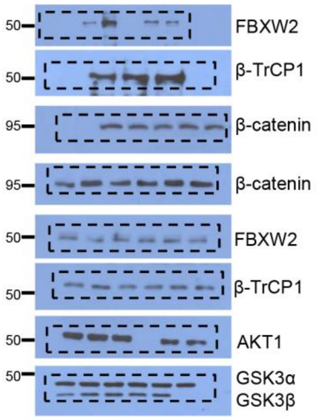

Supplementary Figure 4d

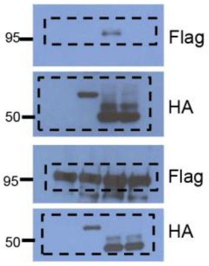

Supplementary Figure 4e

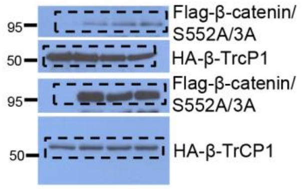

Supplementary Figure 4f

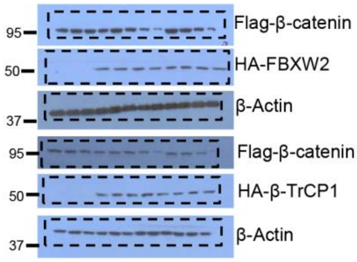

Supplementary Figure 4g

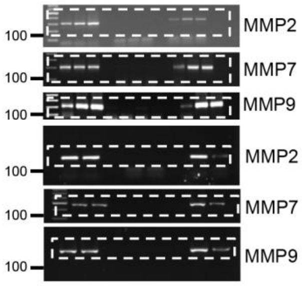

Supplementary Figure 7b

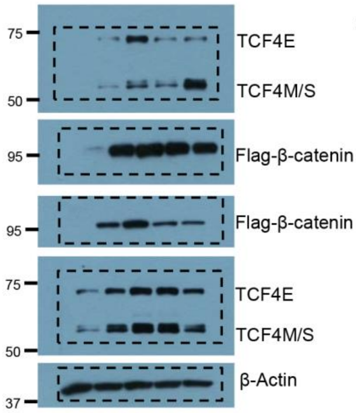

Supplementary Figure 7c

Supplementary Table 1. List of primers used for mutagenesis

Target Gene	Forward primer	Reverse primer
β-catenin-S552A	ACCCAGCGCCGTACGGCCATGGGTGGGACACAG	CTGTGTCCCACCCATGGCCGTACGGCGCTGGGT
β-catenin-3A	GCGGCCATGGGTGGGGCACAGCAGCAATTTGTGGAG	CTCCACAAATTGCTGCTGTGCCCCACCCATGGCCGC
β-catenin-S552D	GATACCCAGCGCCGTACGGACATGGGTGGGACACAG	CTGTGTCCCACCCATCATATCACGGCGCTGGGTATC
β-catenin-3D	GATGACATGGGTGGGGATCAGCAGCAATTTGTGGAG	CTCCACAAATTGCTGCTGATCCCCACCCATGTCATC

Supplementary Table 2. List of siRNAs and shRNAs

Target Gene	sequence
siβ-TrCP1-1	GCGTTGTATTCGATTTGATAA
siβ-TrCP1-2	AAG TGGAATTTGTGGAACATC
siFBXW2-1	GCCTTTGAAACCTCGTCATTA
siFBXW2-2	GCAGCGGTGAAGTTTGATGAA
siMMP2	GCTGAAGGACACACTAAAGAA
siMMP9	CATTCAGGGAGACGCCCATTT
sh-FBXW2-1	CCGGGCCTTTGAAACCTCGTCATTACTCG AGTAATGACGAGGTTTCAAAGGCTTTTTG
sh-FBXW2-2	CCGGGCAGCGGTGAAGTTTGATGAACTCG AGTTCATCAAACTTCACCGCTGCTTTTTG
sh-β-catenin	CCGGTTGTTATCAGAGGACTAAATACTCG AGTATTTAGTCCTCTGATAACAATTTTTG.

Supplementary Table 3. List of primers used for ChIP qPCR

**Target Gene**	**Forward primer**	**Reverse primer**
MMP2	GAGGTCGCTTTCTTTGCCATCT	AGCGACTCCATCTTGAACAGG
MMP7	TTGTGTGCTTCCTGCCAATA	TTGGACCTATGGTTGATTTGG’
MMP9	CGGCATCGGGCAGGGTCT	CACTGTATCCTTGACCTTCTTTCTGG
MYC	TTGCTGGGTTATTTTAATCAT	ACTGTTTGACAAACCGCATCC
CCND1	TGCTTAACAACAGTAACGT	GGGGCTCTTCCTGGGCAGC

Supplementary Table 4. List of primers used for real-time qPCR

**Gene symbol**	**Accession no.**	**Forward primer**	**Reverse primer**
CTNNB1	NM_001904.4	TACAACTGTTTTGAAAATCCA	CGAGTCATTGCATACTGTCC
MMP2	NM_004530.6	CTGCATCCAGACTTCCTCAG	TCCTGGCAATCCCTTTGTATGT
MMP7	NM_002423.5	GGTCACCTACAGGATCGTATCATAT	GGGATCTCTTTGCCCCACAT
MMP9	NM_004994.3	CGCAGACATCGTCATCCAGT	AACCGAGTTGGAACCACGAC
MYC	NM_001354870.1	CTTCTCTCCGTCCTCGGATTCT	GAAGGTGATCCAGACTCTGACCTT
CCND1	NM_053056.2	ATGTTCGTGGCCTCTAAGATGA	CAGGTTCCACTTGAGCTTGTTC
ATCB	NM_001101.5	TCACCCACACTGTGCCCATCTAC	GGAACCGCTCATTGCCAATG
