# Supplementary material for: FBXW2 suppresses migration and invasion of lung cancer cells via promoting β-catenin ubiquitylation and degradation
Source: Nat Commun. 2019 Mar 27;10:1382. doi: 10.1038/s41467-019-09289-5 (PMC6437151; doi:10.1038/s41467-019-09289-5)
Supplement: Supplementary file 3 — Reporting Summary [file 41467_2019_9289_MOESM3_ESM.pdf]

nature research

Corresponding author(s): Yi Sun NCOMMS-18-28100-T

Last updated by author(s): 2/14/2019

Reporting Summary

Nature Research wishes to improve the reproducibility of the work that we publish. This form provides structure for consistency and transparency in reporting. For further information on Nature Research policies, see Authors & Referees and the Editorial Policy Checklist.

Statistics

For all statistical analyses, confirm that the following items are present in the figure legend, table legend, main text, or Methods section.

n/a	Confirmed	
☐	☒	The exact sample size (*n*) for each experimental group/condition, given as a discrete number and unit of measurement
☐	☒	A statement on whether measurements were taken from distinct samples or whether the same sample was measured repeatedly
☐	☒	The statistical test(s) used AND whether they are one- or two-sided<br>*Only common tests should be described solely by name; describe more complex techniques in the Methods section.*
☒	☐	A description of all covariates tested
☒	☐	A description of any assumptions or corrections, such as tests of normality and adjustment for multiple comparisons
☐	☒	A full description of the statistical parameters including central tendency (e.g. means) or other basic estimates (e.g. regression coefficient) AND variation (e.g. standard deviation) or associated estimates of uncertainty (e.g. confidence intervals)
☐	☒	For null hypothesis testing, the test statistic (e.g. *F*, *t*, *r*) with confidence intervals, effect sizes, degrees of freedom and *P* value noted<br>*Give P values as exact values whenever suitable.*
☒	☐	For Bayesian analysis, information on the choice of priors and Markov chain Monte Carlo settings
☒	☐	For hierarchical and complex designs, identification of the appropriate level for tests and full reporting of outcomes
☐	☒	Estimates of effect sizes (e.g. Cohen's *d*, Pearson's *r*), indicating how they were calculated

Our web collection on statistics for biologists contains articles on many of the points above.

Software and code

Policy information about availability of computer code

Data collection	No software was used.
Data analysis	We used Image-Pro Plus 6.0 (http://www.mediacy.com/imageproplus) and ImageJ 1.48v for photographs analysis and GraphPad Prism 8.0 (https://www.graphpad.com/) for statistical analysis.

For manuscripts utilizing custom algorithms or software that are central to the research but not yet described in published literature, software must be made available to editors/reviewers. We strongly encourage code deposition in a community repository (e.g. GitHub). See the Nature Research guidelines for submitting code & software for further information.

Data

Policy information about availability of data

All manuscripts must include a data availability statement. This statement should provide the following information, where applicable:

- Accession codes, unique identifiers, or web links for publicly available datasets
- A list of figures that have associated raw data
- A description of any restrictions on data availability

The authors declare that all the other data supporting the findings of this study are available within the paper and its supplementary information files, and from the corresponding author upon reasonable request. Gel source images for Figs. 1-4, 6 and Supplementary Figures 1-4, 7 are available in Supplementary Figure 9. A reporting summary for this Article is available as a Supplementary Information file.

Field-specific reporting

Please select the one below that is the best fit for your research. If you are not sure, read the appropriate sections before making your selection.

☒ Life sciences ☐ Behavioural & social sciences ☐ Ecological, evolutionary & environmental sciences

For a reference copy of the document with all sections, see nature.com/documents/nr-reporting-summary-flat.pdf

Life sciences study design

All studies must disclose on these points even when the disclosure is negative.

Sample size	This is relevant to only the mouse studies. No sample size calculations were performed. The number of mice used was based on our experience with the model. The sample sizes were sufficient as we reached statistical significance.
Data exclusions	Criteria for exclusion: illness or poor conditions due to reasons other than treatment; not within 95% range of normal distribution. All criteria were pre-established.
Replication	Three independent experiments were performed to verify the reproducibility of every experimental findings.
Randomization	All mice were randomly divided into different groups.
Blinding	All investigators were blinded to group allocation during data collection and analysis.

Behavioural & social sciences study design

All studies must disclose on these points even when the disclosure is negative.

Study description	*Briefly describe the study type including whether data are quantitative, qualitative, or mixed-methods (e.g. qualitative cross-sectional, quantitative experimental, mixed-methods case study).*
Research sample	*State the research sample (e.g. Harvard university undergraduates, villagers in rural India) and provide relevant demographic information (e.g. age, sex) and indicate whether the sample is representative. Provide a rationale for the study sample chosen. For studies involving existing datasets, please describe the dataset and source.*
Sampling strategy	*Describe the sampling procedure (e.g. random, snowball, stratified, convenience). Describe the statistical methods that were used to predetermine sample size OR if no sample-size calculation was performed, describe how sample sizes were chosen and provide a rationale for why these sample sizes are sufficient. For qualitative data, please indicate whether data saturation was considered, and what criteria were used to decide that no further sampling was needed.*
Data collection	*Provide details about the data collection procedure, including the instruments or devices used to record the data (e.g. pen and paper, computer, eye tracker, video or audio equipment) whether anyone was present besides the participant(s) and the researcher, and whether the researcher was blind to experimental condition and/or the study hypothesis during data collection.*
Timing	*Indicate the start and stop dates of data collection. If there is a gap between collection periods, state the dates for each sample cohort.*
Data exclusions	*If no data were excluded from the analyses, state so OR if data were excluded, provide the exact number of exclusions and the rationale behind them, indicating whether exclusion criteria were pre-established.*
Non-participation	*State how many participants dropped out/declined participation and the reason(s) given OR provide response rate OR state that no participants dropped out/declined participation.*
Randomization	*If participants were not allocated into experimental groups, state so OR describe how participants were allocated to groups, and if allocation was not random, describe how covariates were controlled.*

Ecological, evolutionary & environmental sciences study design

All studies must disclose on these points even when the disclosure is negative.

Study description	*Briefly describe the study. For quantitative data include treatment factors and interactions, design structure (e.g. factorial, nested, hierarchical), nature and number of experimental units and replicates.*
Research sample	*Describe the research sample (e.g. a group of tagged Passer domesticus, all Stenocereus thurberi within Organ Pipe Cactus National Monument), and provide a rationale for the sample choice. When relevant, describe the organism taxa, source, sex, age range and any manipulations. State what population the sample is meant to represent when applicable. For studies involving existing datasets,*

describe the data and its source.

Sampling strategy	Note the sampling procedure. Describe the statistical methods that were used to predetermine sample size OR if no sample-size calculation was performed, describe how sample sizes were chosen and provide a rationale for why these sample sizes are sufficient.
Data collection	Describe the data collection procedure, including who recorded the data and how.
Timing and spatial scale	Indicate the start and stop dates of data collection, noting the frequency and periodicity of sampling and providing a rationale for these choices. If there is a gap between collection periods, state the dates for each sample cohort. Specify the spatial scale from which the data are taken
Data exclusions	If no data were excluded from the analyses, state so OR if data were excluded, describe the exclusions and the rationale behind them, indicating whether exclusion criteria were pre-established.
Reproducibility	Describe the measures taken to verify the reproducibility of experimental findings. For each experiment, note whether any attempts to repeat the experiment failed OR state that all attempts to repeat the experiment were successful.
Randomization	Describe how samples/organisms/participants were allocated into groups. If allocation was not random, describe how covariates were controlled. If this is not relevant to your study, explain why.
Blinding	Describe the extent of blinding used during data acquisition and analysis. If blinding was not possible, describe why OR explain why blinding was not relevant to your study.

Did the study involve field work? ☐ Yes ☒ No

Reporting for specific materials, systems and methods

We require information from authors about some types of materials, experimental systems and methods used in many studies. Here, indicate whether each material, system or method listed is relevant to your study. If you are not sure if a list item applies to your research, read the appropriate section before selecting a response.

Materials & experimental systems

n/a	Involved in the study
☐	☒ Antibodies
☐	☒ Eukaryotic cell lines
☒	☐ Palaeontology
☐	☒ Animals and other organisms
☒	☐ Human research participants
☒	☐ Clinical data

Methods

n/a	Involved in the study
☒	☐ ChIP-seq
☒	☐ Flow cytometry
☒	☐ MRI-based neuroimaging

Antibodies

Antibodies used

β-catenin (Santa Cruz, sc-59737), phospho-β-catenin (Ser552) (Cell Signaling, #5651), FBXW2 (Abcam, ab83467; Proteintech, #11499-1-AP), SKP2 (Cell Signaling, #2652), β-TrCP1 (Cell Signaling ,#4394), HA (Roche Life Science, #11867423001), FLAG (Sigma, #F1804), β-Actin (Santa Cruz, sc-47778), FBXL11 (Santa Cruz , sc-135126), GSK3β (Cell Signaling, #12456), AKT (Cell Signaling, #4691), phospho-AKT1 (Ser473) (Cell Signaling, #4051), PARP (Cell Signaling, #9532), caspase3 (Santa Cruz, sc-7272), phospho-β-catenin (Ser33/37Thr41) (Cell Signaling, #9561) and TCF4 (Cell Signaling, #2569).

Validation

1. mouse-β-catenin (Santa Cruz, sc-59737; 1:1000 overnight, 4 ºC for IB; 1:400 overnight, 4 ºC for IHC) Ref：J Cell Biochem. 2017 Dec;118(12):4425-4435.
2.rabbit-phospho-β-catenin (Ser552) (Cell Signaling, #5651, 1:1000 overnight, 4 ºC for IB) Ref：JCI Insight. 2017 Sep 7;2(17). pii: 94904.
3.rabbit-FBXW2 (Proteintech, #11499-1-AP,1:1000 overnight, 4 ºC for IB;1:100 overnight, 4 ºC for IHC) Ref：Nat Commun. 2017 Jan 16;8:14002.
4.rabbit-SKP2(Cell Signaling, #2652, 1:1000 overnight, 4 ºC for IB) Ref：Nat Commun. 2017 Jan 16;8:14002.
5.rabbit-β-TrCP1 (Cell Signaling ,#4394, 1:1000 overnight ,4 ºC for IB) Ref：Nat Commun. 2017 Jan 16;8:14002.
6.rat-HA (Roche Life Science, #11867423001,1:1000 overnight, 4 ºC for IB) Ref：EMBO J. 2011 Mar 2;30(5):823-34.
7.mouse-FLAG (Sigma, #F1804, 1:1000 overnight, 4 ºC for IB) Ref：Nucleic Acids Res. 2017 Apr 20;45(7):4189-4201.
8.mouse-β-Actin (Santa Cruz, sc-47778, 1:1000 overnight, 4 ºC for IB) Ref：Neuropharmacology. 2018 Jan;128:340-350.
9.rabbit-FBXL11 (Santa Cruz , sc-135126, 1:1000 overnight, 4 ºC for IB) Ref：Nat Commun. 2017 Jan 16;8:14002.
10.rabbit-GSK3β (Cell Signaling, #12456, 1:1000 overnight, 4 ºC for IB) Ref：Mol Cell. 2011 Oct 21;44(2):304-16.
11.rabbit-AKT (Cell Signaling, #4691, 1:1000 overnight, 4 ºC for IB) Ref：BMC Biol. 2018 Aug 17;16(1):92.
12.rabbit-phospho-AKT1 (Ser473) (Cell Signaling, #4051, 1:1000 overnight, 4 ºC for IB) Ref：Cancers (Basel). 2018 May 18;10(5).
13.rabbit-PARP (Cell Signaling, #9532,1:1000 overnight, 4 ºC for IB) Ref：Cell Res. 2018 Jul;28(7):756-770.
14.rabbit-caspase3 (Santa Cruz, sc-7272, 1:1000 overnight, 4 ºC for IB) Ref：Cell Biol Toxicol. 2018 Feb;34(1):7-21.
15. rabbit-phospho-β-catenin (Ser33/37Thr41) (Cell Signaling, #9561, 1:1000 overnight, 4 ºC for IB) Ref：Nat Commun. 2018 9:1920.
16. rabbit-TCF4 (Cell Signaling, #2569, 1:1000 overnight, 4 ºC for IB) Ref：Nat Commun. 2018 9:1920.

Eukaryotic cell lines

Policy information about cell lines

Cell line source(s)	All the cell lines derived from human used in the manuscript were obtained from American Type Culture Collection (ATCC) .H1299 (ATCC® CRL-5803™), A549 (ATCC® CCL-185™), NCI-H358 [H-358, H358] (ATCC® CRL-5807™) ,293 [HEK-293] (ATCC® CRL-1573™). Primary MEF cells were generated by our lab.
Authentication	Some of the cell lines used were authenticated.
Mycoplasma contamination	All the cell lines used were negative for mycoplama contamination.
Commonly misidentified lines (See ICLAC register)	No cell lines used in this manuscript were misidentified.

Animals and other organisms

Policy information about studies involving animals; ARRIVE guidelines recommended for reporting animal research

Laboratory animals	Five - to six-week-old BALB/c athymic nude mice (nu/nu, female) were used.
Wild animals	This study did not involve wild animals.
Field-collected samples	This study did not involve samples collected from the field.
Ethics oversight	Mouse experiments were approved by the Review Committee of Zhejiang University School of Medicine.

Note that full information on the approval of the study protocol must also be provided in the manuscript.
